# Peripheral immune and metabolic regulation of Aβ and Tau by exercise in Alzheimer’s disease

**DOI:** 10.3389/fimmu.2025.1678526

**Published:** 2025-10-15

**Authors:** Yuehan Yu, Kang Chen

**Affiliations:** ^1^ Physical, Aesthetic and Labor Education Centre, Zhejiang Shuren University, Hangzhou, China; ^2^ Tianjin Key Laboratory of Exercise Physiology and Sports Medicine, Tianjin University of Sport, Tianjin, China

**Keywords:** Alzheimer’s disease, exercise, amyloid-β, Tau homeostasis, peripheral clearance, myokines

## Abstract

Alzheimer’s disease (AD), characterized by the pathological accumulation of amyloid-β (Aβ) and hyperphosphorylated Tau proteins, remains a major global health challenge with limited therapeutic options. Recent findings highlight that peripheral immune and metabolic pathways play a pivotal role in regulating brain Aβ and Tau homeostasis, particularly in response to physical exercise. In this review, we comprehensively examine current clinical and preclinical evidence on how exercise modulates peripheral immune responses, metabolic states, and systemic clearance mechanisms—including hepatic, renal, immune, and glymphatic pathways. We discuss how regular exercise suppresses peripheral inflammation, enhances immune cell–mediated phagocytosis, improves metabolic resilience, and promotes the elimination of neurotoxic proteins. Furthermore, exercise-induced peripheral mediators, such as myokines, non-coding RNAs, and lactate, are shown to mediate inter-organ communication and signaling pathway crosstalk and contribute to neuroprotection. This integrative perspective underscores the therapeutic promise of exercise as a non-pharmacological intervention that targets peripheral immune-metabolic networks to mitigate AD pathology.

## 1 Introduction

Alzheimer’s disease (AD) is the most common neurodegenerative disorder globally, accounting for approximately 60–70% of all dementia cases (1). Currently affecting over 55 million people worldwide, the prevalence of AD is expected to reach 152 million by 2050, presenting significant public health and socioeconomic challenges (2). The hallmark features of AD include the abnormal accumulation of β-amyloid (Aβ) plaques and neurofibrillary tangles (NFTs) formed by hyperphosphorylated Tau protein (3, 4). These pathologies jointly contribute to neuronal damage, synaptic impairment, and cognitive decline. Evidence increasingly indicates that Aβ and Tau interactions amplify their harmful effects, with disruptions in their balance critically driving disease progression (5, 6). Current treatments, such as cholinesterase inhibitors, offer limited symptomatic relief (7). Therapeutic strategies directly targeting Aβ or Tau have also shown disappointing clinical outcomes (8, 9). For example, monoclonal antibodies against Aβ, including aducanumab and lecanemab, provide only modest cognitive improvements in early-stage patients and frequently result in amyloid-related imaging abnormalities (ARIA), which manifest as cerebral edema (ARIA-E) or microhemorrhages (ARIA-H) that may cause neurological complications, raising concerns about their efficacy and safety (10). Similarly, interventions targeting Tau pathology have yet to deliver meaningful clinical success (11). These setbacks highlight the complexity of AD and underscore the urgent need for innovative therapeutic strategies. Recent research emphasizes that regulation of Aβ and Tau is not confined solely to the brain, suggesting that peripheral organs significantly influence these proteins’ dynamics (12, 13), although these mechanisms remain largely unexplored.

Physical exercise has emerged as a promising non-pharmacological intervention for slowing cognitive decline in AD, especially in early stages such as mild cognitive impairment (14, 15). The beneficial effects of exercise largely result from bioactive substances released by contracting muscles—termed myokines—including interleukin-6 (IL-6), irisin, lactate, and non-coding RNAs (16). These molecules circulate systemically, influencing both peripheral organs and the central nervous system(CNS), thereby enhancing neuronal function and structural resilience. Within the brain, exercise increases brain-derived neurotrophic factor (BDNF) expression, which in turn mediates the activation of synaptic plasticity pathways and promotes neurogenesis (17, 18). Additionally, exercise influences peripheral systems by improving hepatic clearance of Aβ, regulating systemic inflammation, and maintaining metabolic balance, which collectively helps sustain Aβ and Tau equilibrium in the brain, delaying neurodegeneration (19). Exercise also reduces peripheral inflammation by suppressing circulating pro-inflammatory cytokines such as TNF-α and IL-1β. Notably, Yang’s study demonstrated this effect through intravenous transfer of plasma from exercise-trained donor rats (20), which reduced neuroinflammation and modulated microglial and astrocytic activity in recipient animals. In addition, exercise enhances clearance-associated proteins such as APOE and low-density lipoprotein receptor-related protein 1 (LRP1) (21). Furthermore, regular physical activity strengthens the integrity of the blood–brain barrier (BBB) by reducing inflammation, enhancing antioxidant defenses, and increasing tight junction protein expression (22). These combined effects limit inflammatory mediator infiltration into the brain, creating an environment conducive to effective clearance of AD–related pathological proteins, including Aβ and Tau, and preventing their excessive production. Amyloidogenesis in AD involves the sequential cleavage of amyloid precursor protein (APP) by β- and γ-secretases, generating Aβ peptides of varying lengths (predominantly Aβ_40_ and Aβ_42_). These peptides initially exist as soluble monomers and oligomers, which are considered the most neurotoxic species due to their ability to disrupt synaptic function and cellular membranes. Over time, these soluble forms aggregate into insoluble fibrils and eventually form the characteristic amyloid plaques. The transition from soluble to insoluble forms represents a critical pathological progression, with soluble oligomers being more diffusible and capable of spreading between brain regions and into the periphery, while insoluble plaques represent end-stage aggregates that are more difficult to clear. Notably, during advanced stages of AD, compromised BBB integrity allows soluble Aβ and Tau to leak into peripheral circulation, where exercise facilitates their removal by enhancing hepatic LRP1 expression and improving renal and lymphatic clearance capacities (23, 24). Collectively, these findings demonstrate that exercise exerts dual protective effects in AD by both modulating central inflammation and boosting peripheral clearance of Aβ and Tau. However, most current studies focus narrowly on specific organs or isolated molecular targets. The comprehensive peripheral–central network through which exercise preserves protein homeostasis in the brain remains poorly defined and warrants detailed investigation.

Here, we systematically evaluate how physical exercise contributes to maintaining brain Aβ and Tau homeostasis through peripheral mechanisms and discuss its potential therapeutic value in AD. Specifically, we examine two primary pathways: first, how exercise mitigates the overproduction of Aβ and Tau by reducing peripheral inflammation and enhancing immunometabolic function, thus modulating critical enzymes such as BACE1; and second, how exercise enhances the functional capacity of key peripheral organs—such as the liver, kidneys, and glymphatic–lymphatic systems—and improves immune-mediated clearance and metabolic regulation. We also highlight signaling mediators induced by exercise, including myokines (e.g., irisin), cytokines (e.g., IL-6, IL-10), and non-coding RNAs (e.g., miR-132, miR-124), that facilitate communication between peripheral systems and the brain. By presenting an integrated framework encompassing interactions among organs, cells, pathways, and molecules, this review provides a comprehensive understanding of the systemic benefits of exercise, laying the groundwork for translating these insights into clinical AD interventions.

## 2 Effects of exercise on cognitive function in AD

Exercise, a structured subtype of physical activity, refers to planned and repetitive movements performed with the goal of improving or maintaining physical health and fitness (25). According to movement patterns, physiological targets, and metabolic demands, exercise is generally categorized into four main types (**Table 1**). Aerobic exercise, characterized by the rhythmic and sustained activation of large muscle groups, improves cardiovascular endurance and includes activities such as jogging, swimming, and cycling (25). Resistance training enhances muscular strength and endurance through external loads, with common examples including weightlifting, push-ups, and squats (25). High-intensity interval training (HIIT) involves alternating bursts of vigorous activity with periods of low-intensity recovery, combining aerobic and anaerobic benefits within a shorter time frame (25). Mind–body exercises, such as Tai Chi and yoga, emphasize coordinated breathing, posture control, and mental focus, and are particularly suitable for older adults due to their low-impact and integrative nature (25). Growing evidence indicates that regular engagement in these exercise modalities not only enhances metabolic and immune function but also significantly slows age-related cognitive decline, ultimately improving quality of life in aging populations and individuals at risk for or diagnosed with AD (26–28).

**Table 1:** Taxonomy of exercise modalities and their physiological characteristics.

Exercise type	Characteristics	Primary physiological benefits	Common settings	Examples	References
Aerobic Exercise	Continuous, rhythmic activity using large muscle groups	Improves cardiovascular and respiratory endurance	Outdoor tracks, gyms, swimming pools	Jogging, swimming, cycling	(25)
Resistance Exercise	Muscle contractions against external resistance	Increases muscular strength, power, and bone density	Gyms, home-based with weights or resistance bands	Weightlifting, push-ups, squats	(25)
High-Intensity Interval Training (HIIT)	Alternating high- and low-intensity efforts	Enhances both aerobic and anaerobic capacity	Fitness studios, sports fields, home routines	Sprint intervals, circuit training	(25)
Mind–Body Exercise	Integration of movement with mental focus and breath control	Promotes balance, flexibility, and stress reduction	Studios, community centers, home environments	Tai Chi, yoga, Pilates	(25)

An increasing body of clinical and preclinical research indicates that exercise exerts domain-specific and selective effects on cognitive function in individuals with AD or those at elevated risk (**Table 2**). Among various cognitive domains, memory—particularly long-term memory and delayed recall—appears to benefit most consistently from exercise interventions. Meta-analyses of multiple randomized controlled trials (RCTs) have demonstrated that both aerobic and resistance training can enhance episodic memory and executive function in populations with AD or mild cognitive impairment (MCI), whereas improvements in working memory, attention, and verbal fluency are more variable and often restricted to the early stages of disease (29, 30). Notably, global cognition, as measured by standardized assessments such as the Mini-Mental State Examination (MMSE) and the Alzheimer’s Disease Assessment Scale–Cognitive Subscale (ADAS-Cog), shows moderate improvement following regular physical exercise, suggesting that exercise may confer broad-spectrum cognitive benefits. Emerging evidence also supports a dose–response relationship between exercise volume and cognitive outcomes. A recent study revealed that the most pronounced cognitive benefits were observed at a moderate exercise dose of approximately 650 metabolic equivalent of task (MET)·minutes per week, which corresponds to around 150 minutes of moderate-intensity aerobic exercise or an equivalent combination of exercise modalities. The trial was conducted in non-demented, physically inactive older adults, suggesting that these findings may be extrapolated to populations at increased risk for AD. Beyond 1000 MET·min/week, cognitive gains tend to plateau, indicating that excessive training does not yield additional benefits (31). Furthermore, a minimum frequency of three sessions per week, combined with appropriate intensity and duration, appears necessary to achieve meaningful cognitive improvement. Among the various exercise modalities, aerobic exercise remains the most consistently effective intervention for enhancing cognitive function in AD (32). However, high-intensity interval training (HIIT) and mind–body exercises, such as Tai Chi, have also shown promise, particularly in improving executive control and cognitive flexibility (33, 34). In terms of intervention durability, cognitive improvements are generally confined to the active intervention period and tend to wane after cessation. Although some studies suggest that benefits may persist for 6–12 months post-intervention (35), sustained engagement in exercise appears essential for maintaining long-term cognitive gains. Epidemiological evidence reinforces this notion, showing that individuals who maintain high levels of physical activity throughout life experience slower cognitive decline and a reduced risk of dementia (32). Animal studies corroborate these findings (36). In transgenic AD mouse models such as APP/PS1, both forced and voluntary exercise training have been shown to improve spatial learning and memory performance—even in the presence of substantial amyloid pathology (37). Importantly, animals that initiated exercise early in the disease course and continued training over time demonstrated greater cognitive benefits (38), highlighting the critical role of intervention timing and duration. Although effect sizes are typically larger in animal models compared to human trials, the two lines of evidence converge in demonstrating similar domain-specific improvements, particularly in memory and executive function. Collectively, these findings underscore the efficacy of regular physical exercise as a behavioral intervention to improve AD-related cognitive impairments. Optimized exercise prescriptions—including tailored intensity, frequency, and duration—are crucial to achieving maximal cognitive benefit. Long-term, individualized exercise programs offer promising potential to enhance functional outcomes in patients with AD and provide a strong scientific rationale for incorporating physical activity into core non-pharmacological treatment strategies for the disease.

**Table 2:** Effects of exercise on AD patients.

Subjects/age (years)	Intervention groups	Exercise protocol	Main effects	References
50 participants with MCI or early-stage AD (aged 45–90 years)	Multimodal lifestyle intervention (including structured physical activity) *vs* usual care	20-week multimodal program comprising moderate-intensity aerobic and behavioral components	Slowed or reversed cognitive decline, as measured by ADAS-Cog and Clinical Dementia Rating (CDR) scales	(39)
23 cognitively unimpaired, middle-aged adults with genetic or familial risk for AD (mean age ~65 years)	Supervised aerobic treadmill training *vs* no-exercise control	26-week aerobic training program on a treadmill, performed under supervision	Increased plasma cathepsin B (CTSB) levels, which correlated with cognitive improvement; no change in klotho; reduced circulating BDNF	(40)
200 community-dwelling older adults with mild AD (mean age ~73 years)	Supervised moderate-to-high intensity aerobic exercise *vs* stretching/toning control	16-week supervised aerobic training (60 min/session, 3 sessions/week)	No significant change in global cognition (Intent-to-treat analysis); reduced neuropsychiatric symptoms (Neuropsychiatric Inventory); exploratory subgroup analysis suggested cognitive benefit (Symbol Digit Modalities Test) in participants with high adherence	(41)
Adults with MCI or mild–moderate AD (aged ~60–85 years)	Supervised physical training (PT) *vs* cognitive training (CT) *vs* usual care	6-month supervised aerobic and resistance training (PT) or cognitive sessions (CT)	Both PT and CT attenuated cognitive decline (MMSE); memory improved in MCI subgroup (+6.9% PT, + 8.5% CT); PT also improved cardiovascular function; no effects on attention or executive function	(42)
51 older adults with mild–moderate AD (mean age ~70 years)	Supervised aerobic exercise *vs* usual care	16-week supervised moderate-intensity aerobic training	No significant changes in whole-brain or regional cerebral blood flow observed via MRI	(43)
95 older adults with mild–moderate AD (age not specified)	Supervised aerobic exercise *vs* stretching control (ADEX trial)	16-week supervised aerobic training (~150 minutes/week)	Increased levels of neuron-derived extracellular vesicle (NDEV) biomarkers including proBDNF, BDNF, and humanin, particularly in APOE ϵ4 carriers; no changes observed in circulating exerkines	(44)
494 older adults with mild–moderate dementia (mean age ~77 years)	Supervised moderate-to-high intensity aerobic and resistance training *vs* usual care	4-month supervised community-based program, followed by supported unsupervised activity	Slight but statistically significant worsening in cognitive performance (ADAS-Cog) at 12 months (mean difference –1.4, p = 0.03); improved physical fitness; no effects on other clinical outcomes	(45)
120 community-dwelling older adults with chronic stroke (≥55 years), without dementia	Supervised aerobic exercise (EX) *vs* cognitive–social enrichment (ENRICH) *vs* usual care	6-month supervised intervention program (either exercise or enrichment)	Exercise group showed significant improvement in ADAS-Cog-Plus and ADAS-Cog scores (≥Minimal Clinically Important Difference) during the intervention period; however, effects were not sustained at 6-month follow-up	(46)
Sedentary older adults with amnestic MCI (aged ~65–75 years)	Supervised moderate–to–high intensity aerobic exercise *vs* low-intensity flexibility and balance training	Moderate–to–high intensity aerobic training (frequency and duration not specified)	Exercise group demonstrated slowed cognitive decline over 12 months compared to control; trend toward a dose–response relationship was observed	(47)
31 older adults with dementia (aged 65–93 years)	Tango therapy *vs* standard physical exercise	3-month supervised intervention, 30 minutes/session, 5 sessions/week	Tango group showed significantly improved gait speed and attenuated decline in functional mobility and activities of daily living; control group declined in mobility	(48)

Previous research on the cognitive effects of exercise in AD has primarily centered on mechanisms within the CNS (16, 49). Extensive evidence indicates that exercise promotes hippocampal neurogenesis and synaptic plasticity, enhances the expression of neurotrophic factors such as BDNF (50), and reduces abnormal accumulation of Aβ and Tau in the brain (23)—effects that collectively contribute to improved learning and memory performance in AD models. However, increasing attention has been directed toward the possibility that exercise-induced cognitive benefits may also arise from its regulation of peripheral mechanisms. Emerging studies have identified peripheral exercise-induced factors as critical mediators of neuroprotection in AD, including systemic blood factors (e.g., circulating exerkines such as clusterin) and gut microbiota–derived metabolites (e.g., short-chain fatty acids), suggesting that these systemic adaptations to exercise may represent a key mechanism underlying its cognitive benefits (15, 51, 52). Recent research has begun to elucidate how exercise modulates peripheral immune responses to alleviate chronic inflammation (53), strengthens BBB integrity, and enhances the clearance of pathological proteins such as Aβ and Tau by stimulating peripheral elimination pathways, thereby reducing their cerebral accumulation. These findings offer new insights into the systemic mechanisms through which exercise exerts its neuroprotective effects and underscore the potential of integrating central and peripheral regulatory networks as a unified strategy for the prevention and treatment of AD.

## 3 Exercise-mediated suppression of Aβ and Tau generation in AD

Long-term, structured physical exercise reduces Aβ plaques and NFTs in transgenic mouse models of AD (54). These pathological changes are accompanied by reductions in neuroinflammatory responses and result in improved cognitive and memory function. Zhang’s study demonstrated that three months of voluntary wheel running in APP/PS1 mice markedly reduced the overall burden and size of Aβ plaques and attenuated hippocampal Tau phosphorylation (55). The same study reported reduced neuronal loss, enhanced neurogenesis in the CA3 region and dentate gyrus, and improved performance in spatial memory tasks (56). Exercise also stimulates the expression of neurotrophic factors that support synaptic integrity and neural resilience. For instance, voluntary physical activity increases the levels of BDNF and glial cell line-derived neurotrophic factor (GDNF) (57), which promote structural and functional plasticity. In a human study, researchers collected blood samples from the radial artery and internal jugular vein during aerobic exercise and found that BDNF concentrations rose approximately threefold compared to resting conditions (58). Notably, 70–80% of circulating BDNF originated from central sources (40). Follow-up experiments in mice demonstrated that aerobic exercise also upregulated BDNF mRNA expression in the hippocampus and cortex following treadmill training (59). These findings indicate that exercise alleviates cognitive decline by simultaneously reducing the accumulation of pathological proteins and enhancing neurotrophic signaling pathways that support plasticity. While many studies report consistent cognitive benefits, not all have observed corresponding reductions in Aβ or phosphorylated tau levels. These inconsistencies may arise from differences in the timing of intervention. Exercise initiated after extensive plaque formation appears less effective in reversing established pathology or restoring cognitive function. In contrast, when introduced in early stages or prior to plaque accumulation, exercise slows the progression of pathology and better preserves cognitive performance. This stage-dependent effect has also been observed in clinical studies. In cognitively unimpaired older adults, higher levels of physical activity are associated with lower plasma and cerebral Aβ burden (42). One clinical trial involving participants with MCI found that six months of aerobic exercise reduced plasma Aβ_1–42_ concentrations by 24% (60). Moreover, the form of exercise may influence therapeutic outcomes. In mouse models, voluntary running has demonstrated superior efficacy over forced treadmill exercise in improving cognitive function (61). Collectively, these results highlight the capacity of exercise to regulate Aβ homeostasis and mitigate the neuropathological cascade associated with AD.

### 3.1 Exercise suppresses peripheral inflammation and limits excessive Aβ and Tau production in the brain

A hallmark of AD pathology is the accumulation of extracellular Aβ plaques and intracellular NFTs formed by hyperphosphorylated tau protein. These aberrant protein aggregates disrupt synaptic function and drive progressive cognitive decline. Emerging evidence has highlighted the critical role of chronic peripheral inflammation in exacerbating AD pathogenesis (62). With aging, the integrity of the BBB declines, allowing peripheral immune mediators to access the CNS more readily. Patients with AD exhibit elevated levels of peripheral immune cells and pro-inflammatory cytokines—such as interleukin-6 (IL-6), IL-17, and tumor necrosis factor-α (TNF-α)—in both cerebrospinal fluid(CSF) and peripheral blood (63). These peripheral signals infiltrate the brain, activate microglia and other resident immune cells, and amplify neuroinflammatory responses. Persistent low-grade systemic inflammation engages key pro-inflammatory signaling pathways, including NF-κB and the NLRP3 inflammasome (64). These pathways upregulate the expression of β-site amyloid precursor protein-cleaving enzyme 1 (BACE1), a critical enzyme in Aβ generation (65). In parallel, pro-inflammatory factors aberrantly activate kinases such as cyclin-dependent kinase 5 (CDK5) and glycogen synthase kinase 3β (GSK3β), promoting pathological tau phosphorylation and tangle formation (66). This inflammatory cascade establishes a self-reinforcing loop in which neuroinflammation and proteinopathy exacerbate one another. Given this mechanistic link, peripheral inflammation is increasingly recognized as a driving force in the progression of AD. Therapeutic strategies targeting inflammatory pathways may offer a promising approach to disrupting the pathological feedback between Aβ and tau accumulation.

Regular physical exercise is widely recognized for its anti-inflammatory effects and its capacity to lower chronic systemic inflammation (67). Long-term adherence to moderate-intensity exercise reduces resting levels of pro-inflammatory cytokines while promoting the expression of anti-inflammatory mediators, as demonstrated in patients with chronic kidney disease (68), although caution is warranted when extrapolating these findings to AD. In clinical populations, exercise interventions have demonstrated promising anti-inflammatory benefits in individuals with MCI. For example, a 12-week program combining moderate-intensity aerobic training with cognitive stimulation led to significant reductions in serum IL-1β, IL-6, and TNF-α, along with concurrent decreases in plasma Aβ_40_ and total tau concentrations (69). These findings suggest a potential link between exercise-induced improvements in systemic inflammation and reductions in AD-related pathological markers. Moreover, consistent physical activity enhances the production of peripheral anti-inflammatory cytokines and, within the CNS, promotes a phenotypic shift of microglia from a pro-inflammatory “M1-type” toward an anti-inflammatory “M2-type” polarization (70). Interestingly, acute bouts of vigorous exercise can transiently increase circulating IL-6 levels. However, this elevation triggers a compensatory release of anti-inflammatory cytokines, such as interleukin-1 receptor antagonist (IL-1ra) and IL-10. Over time, this dynamic regulatory cycle contributes to the downregulation of key inflammatory pathways, including NF-κB, ultimately resulting in sustained reductions in basal levels of inflammatory markers such as C-reactive protein (CRP), TNF-α, and IL-6.

**Figure 1:**
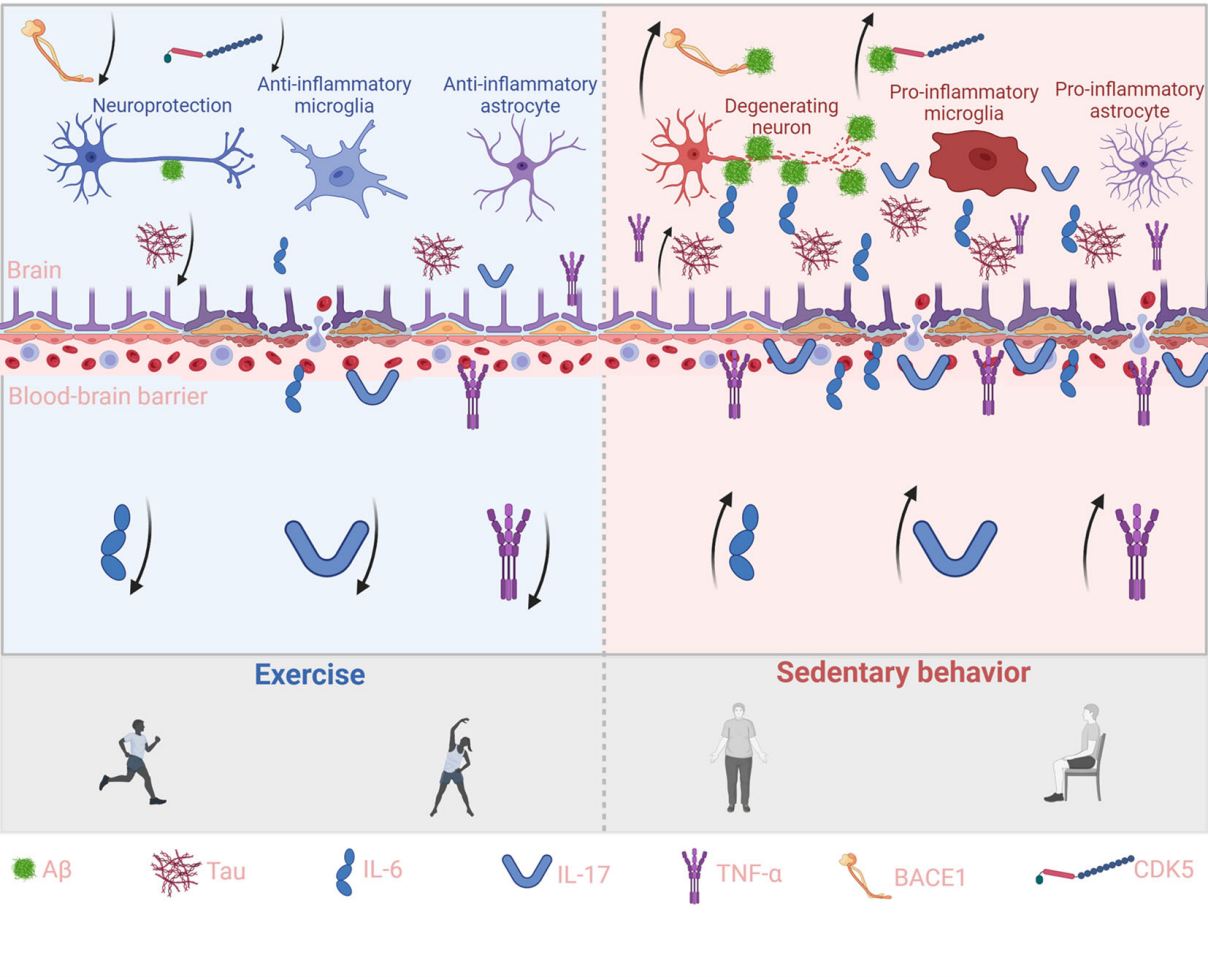
Exercise reduces peripheral cytokines and curbs brain Aβ and Tau accumulation. This schematic illustrates the contrasting effects of exercise (left, blue background) and sedentary behavior (right, red background) on peripheral cytokine signaling, blood–brain barrier (BBB) integrity, and brain pathology. Exercise enhances IL-6 and IL-17 signaling while suppressing pro-inflammatory cytokines such as TNF-α, thereby strengthening BBB function, reducing Aβ and Tau accumulation, and supporting neuroprotection through anti-inflammatory microglia and astrocytes. In contrast, sedentary behavior decreases protective cytokines and elevates pro-inflammatory mediators, impairing BBB integrity, aggravating Aβ and Tau deposition, and driving neurodegeneration through pro-inflammatory glial activation. Key molecules (Aβ, Tau, IL-6, IL-17, TNF-α, BACE1, and CDK5) are indicated at the bottom, and arrows represent the direction of molecular or cellular effects.

In AD, disruption of the BBB is both a contributing factor and a driver of disease progression (71). Compromise of BBB integrity and selective permeability allows microbial metabolites and circulating immunoglobulins to infiltrate the brain parenchyma, where they activate microglia and central immune pathways. Activated microglia trigger the nuclear translocation of NF-κB, leading to the upregulation of pro-inflammatory cytokines such as IL-1β and IL-18 (72–75). This cascade accelerates Aβ deposition and tau phosphorylation, thereby exacerbating neurodegeneration. Microvascular endothelial damage and increased BBB permeability are common in AD and are closely associated with chronic systemic inflammation (76). Regular physical exercise has been shown to protect BBB function and reestablish the immune barrier of the CNS. In animal models, exercise promotes the expression of tight junction proteins in cerebral endothelial cells and increases neurotrophic support to stabilize the vascular endothelium, thereby reducing BBB permeability (77). Exercise also restores the number and activity of astrocytes that are closely associated with the neurovascular unit (78). These astrocytes, often reduced in AD models, are essential for maintaining BBB structure and function. By preserving BBB integrity, exercise limits the entry of peripheral immune mediators into the brain. This restricts the influx of circulating pro-inflammatory cytokines and reduces the recruitment of peripheral immune cells that would otherwise perpetuate neuroinflammation. One study demonstrated that plasma obtained from exercise-trained donor mice suppressed complement-mediated inflammatory responses in the hippocampus of sedentary recipient AD model mice, with clusterin identified as a key mediator. In parallel, physically active human participants showed elevated plasma clusterin levels after exercise training, suggesting that systemic adaptations to exercise may exert remote immunomodulatory effects within the brain (15). Importantly, reduced peripheral inflammation lowers the overactivation of central immune cells, thereby preventing the upregulation of Aβ-generating enzymes. For instance, voluntary wheel running significantly downregulated BACE1 gene expression in the hippocampus of AD mice and was accompanied by reduced levels of soluble Aβ_1–42_ (79). This effect is partly mediated by lactate produced during skeletal muscle contraction, which activates the Sirt1 pathway and enhances BDNF expression in the hippocampus (80), supporting neuronal viability and synaptic plasticity. BDNF not only promotes synaptic remodeling but also suppresses BACE1 activity, thus inhibiting Aβ generation via the β-/γ-secretase pathway (81). In addition to reducing Aβ burden, exercise helps mitigate tau pathology by modulating inflammation-associated kinase activation. Chronic inflammation has been shown to activate tau kinases such as GSK3β and CDK5 (82), which play pivotal roles in hyperphosphorylation and neurofibrillary tangle formation. Exercise dampens these pathways by suppressing peripheral inflammatory triggers and restoring BBB homeostasis. Furthermore, it activates pro-survival signaling cascades within neurons. In a male rat model of post-traumatic stress disorder (PTSD), moderate exercise was shown to increase the expression of insulin-like growth factor-1 (IGF-1) and activate the BDNF/TrkB axis, which in turn stimulates the PI3K/Akt pathway (83). Akt activation leads to the phosphorylation and inhibition of GSK3β, thereby reducing its capacity to phosphorylate tau. Supporting this mechanism, treadmill exercise in animal models elevates hippocampal p-Akt levels and concurrently reduces active GSK3β and tau phosphorylation (84). Similarly, voluntary running activated the BDNF/TrkB/Akt axis and promoted GSK3β inactivation, alleviating tau pathology (85). The anti-inflammatory effects of exercise also prevent sustained activation of neurotoxic kinases. For example, chronically activated microglia release IL-1β and other cytokines that accelerate tau propagation and aggregation (86). Exercise has been shown to reduce IL-1β levels and suppress pro-inflammatory microglial phenotypes. In a short-term resistance training study, 3xTg-AD mice subjected to ladder-climbing exercise exhibited reduced Aβ load and tau phosphorylation (87), attenuated glial activation, and improved synaptic function. Together, these findings demonstrate that exercise reduces peripheral pro-inflammatory signaling and strengthens BBB function, forming a defensive barrier against systemic immune insults. By reshaping the brain’s inflammatory microenvironment, exercise slows Aβ and tau production and ultimately alleviates AD-related pathology. In sum, structured physical training reprograms the peripheral immune system and shifts the central immune tone from a pro-inflammatory to an anti-inflammatory state, offering systemic immunoregulatory support that may help delay disease progression in AD (**Figure 1**).

### 3.2 Exercise modulates gut microbiota dysbiosis to reduce aberrant Aβ and Tau accumulation in the brain

The gut microbiota significantly influences CNS health by shaping immune responses, neuronal function, and glial cell activity through the gut–brain axis, with microbiota-derived metabolites such as short-chain fatty acids (e.g., acetate, propionate, butyrate), tryptophan-derived indoles, and secondary bile acids serving as key mediators of this communication (88). In AD, disrupted gut microbiota, known as dysbiosis, weakens intestinal barrier integrity, leading to increased permeability and systemic inflammation (89). This heightened inflammatory state exacerbates neuroinflammation and accelerates AD pathology, creating a self-reinforcing cycle. Notably, AD patients exhibit reduced microbial diversity characterized by increased levels of harmful bacteria such as Proteobacteria and Bacteroidetes, along with decreased beneficial groups like Firmicutes and Bifidobacterium (90). These changes lower the production of short-chain fatty acids (SCFAs), especially butyrate, impairing protective mechanisms like BDNF signaling and facilitating Tau protein abnormalities. Moreover, pathogenic bacteria contribute to AD pathology primarily through endotoxins such as lipopolysaccharide (LPS) (91), which induce neuroinflammation and exacerbate Aβ accumulation, Tau hyperphosphorylation, and blood–brain barrier disruption, thereby accelerating cognitive decline.

**Figure 2:**
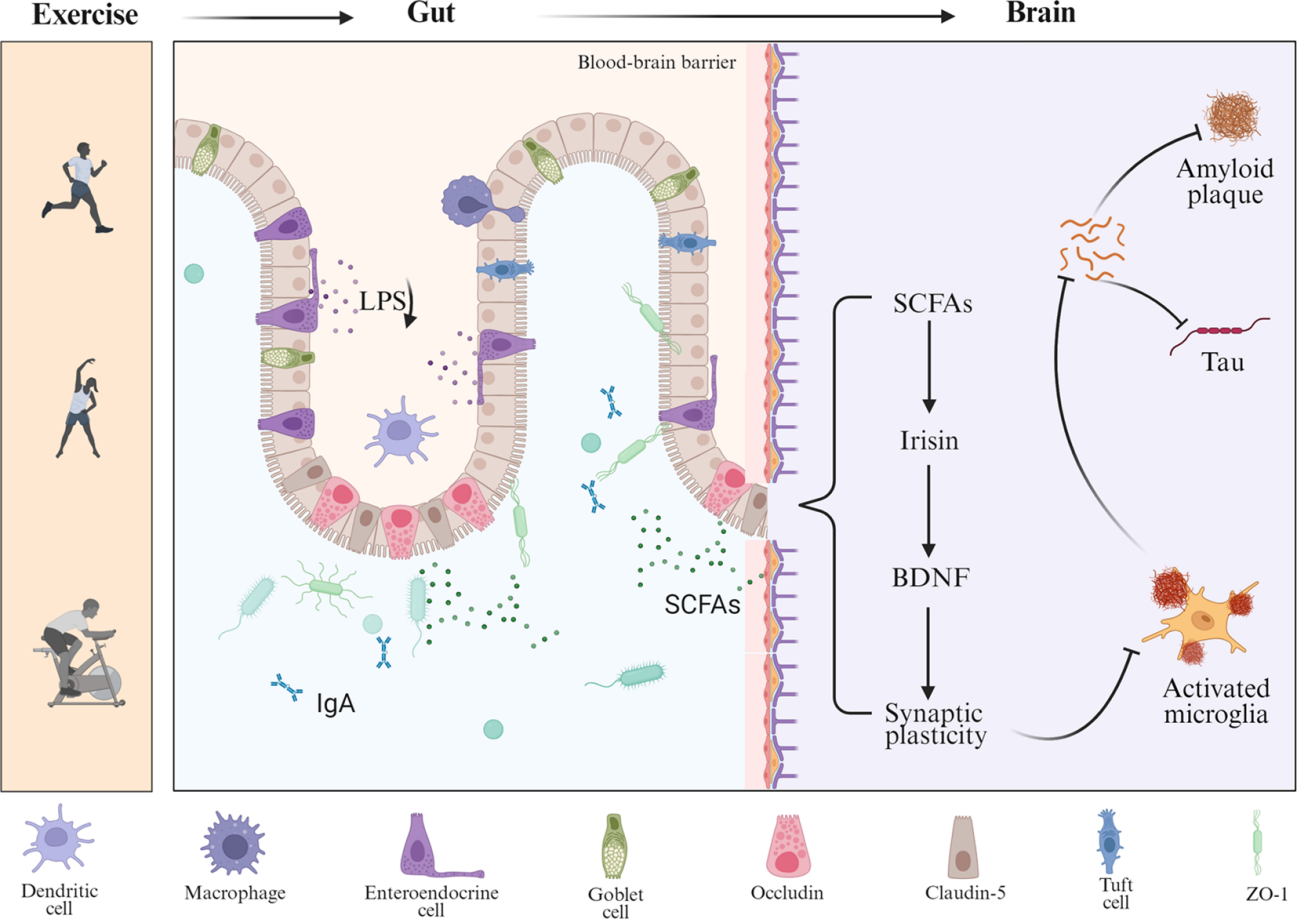
Multilevel regulation of Aβ and Tau pathology by exercise through the gut–brain axis. This schematic illustrates how exercise modulates the gut–brain axis to influence AD pathology. Exercise alters gut microbial composition, increasing the production of SCFAs and reducing harmful metabolites such as LPS, while also enhancing protective immune factors like IgA. These changes strengthen intestinal barrier integrity through tight junction proteins (occludin, claudin-5, and ZO-1) and reduce peripheral inflammation. Circulating SCFAs act on the brain, where they promote the release of myokines such as irisin and upregulate BDNF, thereby enhancing synaptic plasticity.

Mounting evidence suggests that exercise reshapes gut microbiota composition and metabolite profiles to reduce the cerebral accumulation of Aβ and Tau proteins. Regular physical activity has been shown to enrich beneficial gut bacteria and enhance the production of SCFAs, such as acetate, propionate, and butyrate (92). These metabolites readily cross the BBB, where they exert anti-inflammatory and neuroprotective effects, with butyrate exhibiting the most pronounced activity. Butyrate—an SCFA acting as a histone deacetylase (HDAC) inhibitor—enhances histone acetylation at neurotrophic gene loci and thereby upregulates BDNF, supporting synaptic plasticity and cognition (93). Recent evidence further shows that butyrate elevates brain BDNF and downstream PI3K/Akt signaling *in vivo (* 94). Experimental studies have shown that butyrate treatment increases phosphorylation at serine-9 and acetylation at lysine-15 of GSK3β, reducing its activity and mitigating Tau hyperphosphorylation and neurofibrillary pathology (95). In parallel, exercise-enhanced microbial metabolism suppresses CNS inflammation through SCFA receptor activation in colonic epithelial cells and by modulating glial cell activity (96, 97). Conversely, exercise selectively decreases pro-inflammatory bacterial populations and their harmful metabolites. Notably, regular exercise lowers circulating levels of LPS (98), an endotoxin produced by Gram-negative bacteria, while enhancing intestinal barrier integrity by upregulating tight junction proteins such as ZO-1, Occludin, and Claudin-5 (22). This prevents LPS translocation from the gut lumen into the bloodstream. In APP/PS1 mice, 12 weeks of treadmill training significantly reduced LPS levels in plasma and brain tissue, improved BBB function, and attenuated microglia-mediated neuroinflammation (98). Moreover, exercise modulates peripheral immune and neuroendocrine pathways to facilitate gut–brain communication, in part by altering gut microbiota composition and enhancing the production of beneficial metabolites such as short-chain fatty acids and tryptophan-derived indoles (99–101). It reduces pro-inflammatory monocyte subsets and cytokine while increasing anti-inflammatory mediators. Long-term training also downregulates TLR4 expression on monocytes, diminishing LPS responsiveness and further limiting systemic inflammation—an effect that may counteract chronic neuroinflammation in AD (102). Systemic exercise-induced factors, termed exerkines, also mediate gut–brain interactions. Irisin, a myokine derived from fibronectin type III domain-containing protein 5 (FNDC5), is upregulated by aerobic exercise and has been shown to enhance hippocampal neuroplasticity and cognition (103). Recent studies suggest that irisin reprograms gut microbial activity and suppresses gut–brain axis inflammation, mitigating aging- and AD-related cognitive decline (104). Furthermore, breathing-based training enhances vagal tone in humans (105), and aerobic/resistance exercise is also associated with increases in vagally mediated HRV (106). Enhanced vagal signaling, in turn, supports gastrointestinal motility and secretion, thereby contributing to microbial homeostasis (107). Notably, BDNF itself plays a dual role in the exercise–gut–brain axis (108). Aerobic exercise markedly increases central and peripheral BDNF levels (109), supporting neuronal survival and synaptic function (110). BDNF deficiency impairs colonic epithelial integrity, as evidenced by reduced expression of ZO-1, Occludin, and Claudin-1 and elevated Claudin-2 (111), along with microvillus degeneration and microbial translocation. Reciprocally, gut microbial dysbiosis can reduce hippocampal BDNF and TrkB expression, as demonstrated in antibiotic-treated animals (112). These findings suggest that BDNF acts both centrally and peripherally to coordinate neuroplasticity, gut barrier integrity, and inflammatory tone in AD. Animal studies provide direct support for this concept. In 3×Tg-AD mice, 20 weeks of treadmill exercise significantly increased Akkermansia muciniphila and reduced pro-inflammatory Bacteroides (113). These microbiota shifts were accompanied by upregulation of BBB proteins, decreased cerebral Aβ and Tau pathology, and improved spatial memory. This suggests that exercise remodels the gut–brain axis to preserve cognition via peripheral barrier reinforcement. Collectively, exercise sustains Aβ and Tau homeostasis through coordinated peripheral mechanisms—including metabolic modulation, immune suppression, and neuroendocrine regulation—thereby offering a multi-targeted and feasible intervention strategy against AD (**Figure 2**).

### 3.3 Exercise enhances energy metabolism to reduce excessive Aβ and Tau accumulation in the brain

Dysregulated energy metabolism is considered one of the central driving forces in the pathogenesis of AD (114). Substantial evidence indicates that cerebral glucose metabolism is already impaired in the early stages of AD, especially in cognition-related regions such as the hippocampus and cortex (115, 116). These impairments, including reduced glucose uptake and diminished energy production, often precede hallmark pathological features such as Aβ deposition and Tau hyperphosphorylation (117). Impaired neuronal glucose utilization results in mitochondrial dysfunction and oxidative stress, which subsequently trigger signaling cascades that enhance Aβ production and Tau phosphorylation. Additionally, peripheral insulin resistance exacerbates central insulin signaling deficits, reduces Aβ clearance by insulin-degrading enzyme (IDE) (118), and activates glycogen synthase kinase-3β (GSK-3β), thereby contributing to the pathological phosphorylation of Tau (119). Epidemiological studies further support this link, showing that type 2 diabetes and other energy-related metabolic disorders substantially increase the risk of developing AD (120, 121). These findings suggest that early intervention targeting both peripheral and central metabolic dysfunction may offer a promising strategy for delaying the onset and progression of AD.

Regular exercise, recognized as an effective lifestyle intervention, markedly enhances skeletal muscle mitochondrial function and glucose metabolism, thereby alleviating insulin resistance (122). Mechanistically, exercise activates the AMPK/PGC-1α signaling axis to promote mitochondrial biogenesis and mitochondrial quality control processes, including fission–fusion dynamics and mitophagy, in skeletal muscle and thereby enhances glucose uptake and utilization (123). This metabolic optimization reduces peripheral hyperinsulinemia and restores systemic insulin sensitivity. These effects further enhance insulin signaling in the brain, inhibit GSK-3β activity (124), and attenuate Tau hyperphosphorylation. Additionally, reduced peripheral insulin levels lessen the competitive inhibition of IDE, thereby facilitating more efficient Aβ clearance. Long-term aerobic exercise significantly decreases hippocampal Aβ deposition and Tau phosphorylation in AD transgenic mice, with mechanisms involving reduced APP phosphorylation, downregulation of γ-secretase Presenilin-1, and GSK-3β inactivation (124). In AD models with glucose metabolism disorders, exercise reverses diabetes-induced cognitive deficits and Tau hyperphosphorylation by inhibiting the FOXO1/NF-κB/NLRP3 inflammatory pathway and activating PI3K/Akt signaling (125, 126). In humans, older adults who consistently engage in physical activity exhibit improved insulin sensitivity, lower fasting insulin and lipid levels, and reduced cerebral Aβ burden (127). Exercise also modulates adipose tissue endocrine function, lowering systemic chronic inflammation and indirectly alleviating Aβ and Tau pathology in the brain. In obesity and insulin-resistant states, excessive secretion of pro-inflammatory cytokines from adipose tissue activates microglial inflammation, upregulates BACE1, and promotes Tau hyperphosphorylation (128). Regular exercise reduces adiposity, suppresses pro-inflammatory cytokine release, and increases adiponectin secretion. Adiponectin, in turn, activates the AMPK/PPARα pathway to enhance systemic glucose and lipid homeostasis while inhibiting NF-κB and IL-6/STAT3 signaling (129). Exercise also shifts adipose macrophages from a pro-inflammatory M1 to an anti-inflammatory M2 phenotype (64), further lowering systemic inflammation and CNS inflammatory stress. Animal studies confirm that these changes, combined with increased anti-inflammatory mediators, reduce brain inflammatory factors, Aβ deposition, and Tau phosphorylation (130). Systemic metabolic improvements are closely synchronized with enhanced brain energy metabolism. Regular exercise increases cerebral blood flow and the transport of nutrients across the BBB (131), directly enhancing glucose availability in the brain. Muscle contractions during exercise release metabolites such as lactate and irisin, which cross the BBB to serve as alternative neuronal energy substrates and activate the cAMP/CREB/BDNF signaling pathway (132), thereby enhancing brain metabolism and neuroplasticity. In AD models, exercise-induced irisin improves synaptic plasticity and cognition while lowering Aβ and Tau pathology (133). In summary, regular exercise mitigates abnormal Aβ and Tau accumulation by enhancing skeletal muscle glucose metabolism and mitochondrial function, modulating adipose endocrine activity to reduce systemic inflammation, and synchronously improving brain energy metabolism.

## 4 Exercise enhances peripheral clearance mechanisms to maintain Aβ and Tau homeostasis

BBB dysfunction is recognized as an early and critical event in the pathogenesis of AD (134). As BBB integrity deteriorates, both Aβ and Tau proteins in the CNS can be transported into the peripheral circulation via passive diffusion or active receptor-mediated mechanisms (135). This enables peripheral organs and systems to participate in the clearance of neurotoxic proteins originating from the brain. Aβ exits the brain through endothelial transport mediated by receptors such as LRP1 and the receptor for advanced glycation end-products (RAGE) (136), while Tau is also detectable in peripheral blood via extracellular vesicles or specific transporters. The integrity of peripheral clearance pathways is thus essential for maintaining cerebral Aβ and Tau homeostasis. However, aging—the most significant risk factor for AD—substantially impairs this defense system. In the elderly, hepatic Aβ uptake declines, renal excretory capacity weakens, and immune-mediated clearance becomes impaired (137). These deficits result in peripheral retention of Aβ and Tau, and potentially their re-entry into the brain, further exacerbating central accumulation and establishing a pathological brain–periphery vicious cycle. Enhancing peripheral clearance has therefore emerged as a promising strategy to mitigate AD pathology. Within this context, exercise—a systemic and modifiable intervention—has shown potential to activate multiorgan clearance mechanisms, including the glymphatic–astrocytic pathway (138), as well as peripheral routes such as the liver, kidney, and immune system (139–141). Exercise increases the expression of key transport receptors and metabolic enzymes in the liver and kidneys, thereby improving organ-level Aβ and Tau clearance (19). It also improves glymphatic fluid dynamics, enhancing cerebrospinal–interstitial fluid exchange and metabolic coupling between the brain and periphery. Furthermore, exercise promotes phenotypic switching and phagocytic capacity of peripheral immune cells (142), offering an additional immunological route for Aβ and Tau clearance. Collectively, these effects suggest that reprogramming peripheral clearance mechanisms may represent a critical pathway through which exercise delays the progression of AD.

### 4.1 Exercise enhances peripheral clearance of Aβ and Tau by modulating immune cell function

The peripheral immune system plays a critical role in maintaining the homeostasis of Aβ and Tau proteins in the brain (119). When the BBB is compromised, brain-derived Aβ and Tau can enter systemic circulation, where they are subject to clearance by immune cells. Recent studies demonstrate that regular exercise enhances peripheral Aβ and Tau clearance by modulating the activation states and functions of monocyte–macrophage cells, neutrophils, and T lymphocytes, thereby contributing to the mitigation of AD pathology (120). Monocyte–macrophage lineage cells are central to the peripheral removal of Aβ and Tau. Under normal conditions, approximately 40–60% of brain Aβ enters the bloodstream and is cleared through this pathway (14). Aging, however, diminishes their phagocytic efficiency, in part due to reduced expression of scavenger receptors such as Toll-like receptor 2 (TLR2) (121). Exercise has been shown to reverse these deficits. In preclinical models, physical activity promotes anti-inflammatory M2 polarization of macrophages and enhances their capacity for Aβ uptake and degradation (122). Exercise also increases Fcγ receptor expression (123), facilitating antibody-dependent phagocytosis and improving overall clearance efficiency. Neutrophils play a complementary role in peripheral protein removal (124). Although their direct phagocytosis of Aβ and Tau is limited, neutrophils release neutrophil extracellular traps that support the capture and degradation of amyloid fibrils by macrophages (143). However, excessive neutrophil activation may exacerbate inflammation and vascular damage. Exercise restores neutrophil homeostasis (125), enhancing their maturation and phagocytic function while limiting pathological activation. These adaptations improve systemic clearance efficiency of brain-derived proteins. T lymphocytes, particularly regulatory T cells (Tregs), may contribute indirectly to immune clearance (126). By secreting anti-inflammatory cytokines such as IL-4 and IL-10, Tregs promote M2 macrophage polarization, which could in turn enhance phagocytic clearance of Aβ and Tau (127). However, this interpretation remains speculative, as direct evidence for Treg-mediated clearance of amyloid pathology is still limited and requires further investigation. In AD, T cell imbalance—characterized by increased Th1/Th17 cells and reduced Treg function—dampens immune tolerance and promotes chronic inflammation (128). Exercise restores T cell subset balance (129), increases Treg prevalence, suppresses pro-inflammatory cytokine expression, and supports anti-inflammatory immune responses. Collectively, exercise-mediated modulation of peripheral immune cells—including enhanced macrophage phagocytosis, functional rebalancing of neutrophils, and T cell–driven immunoregulation—forms a key mechanism through which physical activity facilitates Aβ and Tau clearance and slows AD progression.

### 4.2 Exercise enhances liver–brain axis function to promote peripheral clearance of Aβ and Tau

Following BBB disruption, brain-derived Aβ and Tau proteins that are not cleared by peripheral immune cells can be transported via the bloodstream to peripheral organs for elimination. Under physiological conditions, approximately 40–60% of brain-derived Aβ is cleared through peripheral mechanisms, with the liver serving as the major site of systemic clearance (19). Hepatocytes and liver sinusoidal endothelial cells express high levels of LRP1 (144), a key receptor responsible for binding and internalizing circulating Aβ for degradation. LRP1-mediated hepatic uptake allows for the clearance of roughly 13.9% of Aβ_42_ and 8.9% of Aβ_40_ per circulatory cycle (19). However, aging and AD progression are associated with downregulation of hepatic LRP1 expression, resulting in impaired Aβ clearance and elevated Aβ levels in both plasma and brain. Conversely, enhancing hepatic LRP1 expression has been shown to lower cerebral Aβ burden and improve cognitive outcomes (19), underscoring the importance of an intact liver–brain axis in maintaining Aβ homeostasis. Similarly, Tau protein can enter peripheral circulation via extracellular vesicles and may be degraded in peripheral organs such as the liver (145). The functional integrity of this peripheral clearance route also influences Tau accumulation in the brain. Regular exercise, as a systemic intervention, improves liver function and enhances its capacity for Aβ elimination (123). Aerobic exercise has been shown in animal models to reduce cerebral Aβ load while accelerating hepatic clearance. For example, in APP/PS1 transgenic mice, 8 weeks of treadmill training improved cognitive and exploratory behavior, reduced brain Aβ deposition, and increased hepatic phagocytic and degradative activity toward Aβ (142). Exercise facilitates peripheral Aβ clearance by modulating key molecular pathways. It elevates soluble LRP1 (sLRP1) levels in plasma, which act as peripheral ‘scavengers’ binding circulating Aβ, and simultaneously upregulates membrane-bound LRP1 expression on hepatocytes, which mediates hepatic uptake and degradation of Aβ. These two forms of LRP1 act in a complementary manner to promote efficient systemic clearance of circulating Aβ (146). In AD models, treadmill training increased LRP1 expression in both liver and hippocampus, suggesting a synergistic enhancement of Aβ clearance across the liver–brain axis. In parallel, exercise also increases the expression of Aβ-degrading enzymes, such as IDE in the liver and neprilysin (NEP) in the hippocampus (147). These enzymatic adaptations enhance peripheral Aβ catabolism, reduce its systemic accumulation, and mitigate the risk of re-entry into the brain.

**Figure 3:**
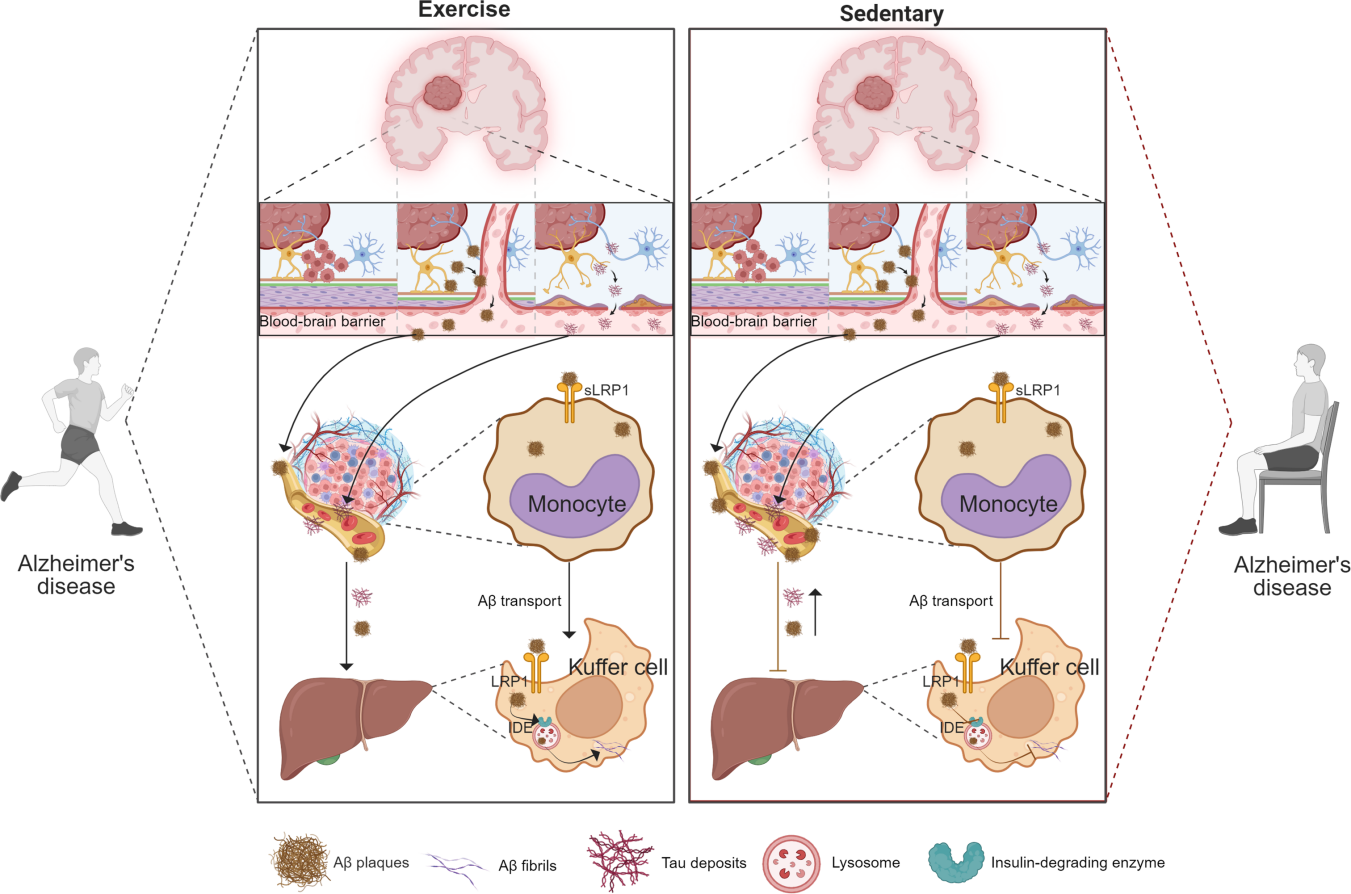
Exercise enhances peripheral clearance of brain-derived Aβ and Tau via monocyte-mediated transport and Kupffer cell degradation. This schematic compares the effects of exercise (left) and sedentary behavior (right) on peripheral clearance pathways for brain-derived proteins. Under exercise conditions, Aβ and Tau are transported across the blood–brain barrier and bound by peripheral monocytes through LRP1-mediated uptake, while Kupffer cells in the liver further degrade these proteins via LRP1 and IDE-dependent mechanisms. These processes collectively enhance systemic elimination of neurotoxic proteins and reduce their accumulation in the brain. In contrast, sedentary behavior is associated with impaired monocyte uptake and reduced Kupffer cell clearance, leading to diminished peripheral removal of Aβ and Tau and their subsequent deposition in the brain. Key molecules (Aβ plaques, Tau tangles, LRP1, IDE) and cellular components (monocytes, Kupffer cells) are indicated in the figure.

Although studies on the peripheral clearance of Tau are currently limited, emerging evidence suggests that the enhanced systemic clearance pathways induced by exercise may also contribute to Tau homeostasis. Tau has been shown to dynamically exchange between the brain and peripheral tissues through the bloodstream, with portions of brain-derived Tau transported to peripheral organs—such as the liver and kidneys—for metabolic degradation (148). A functional peripheral clearance system helps minimize peripheral accumulation and reduces the risk of Tau re-entry into the CNS. Accordingly, improvements in liver–brain axis efficiency induced by exercise may simultaneously enhance the peripheral elimination of Tau. Nwoko’s study demonstrated that aerobic exercise reduced Tau phosphorylation and attenuated Tau-related pathology in AD model mice, potentially due in part to improved clearance of circulating Tau exported from the brain (149). Nevertheless, compared to Aβ, Tau appears to be cleared through more complex peripheral mechanisms, including extracellular vesicle–mediated transport, hepatic macrophage degradation, and renal filtration—all of which require further investigation. In summary, exercise activates multiple mechanisms within the liver–brain axis to promote the peripheral clearance of Aβ and Tau proteins. These include enhanced hepatic uptake and degradation, as well as systemic improvements in liver metabolic health that support sustained protein elimination (**Figure 3**).

### 4.3 Exercise enhances the kidney–brain axis to promote peripheral clearance of Aβ and Tau

In addition to the liver, the kidneys—critical metabolic and excretory organs—play an essential role in the peripheral clearance of circulating Aβ and Tau proteins (150). Under normal physiological conditions, free Aβ (approximately 4 kDa) is filtered through the glomerular barrier into the primary urine and excreted via the urinary tract (151). This has been confirmed in animal tracer studies and through the detection of Aβ in human urine samples. The kidneys also eliminate Aβ through receptor-mediated uptake and enzymatic degradation by renal tubular epithelial cells. These cells highly express megalin (also known as LRP2), a receptor that binds and reabsorbs filtered Aβ, preventing its excessive urinary loss and directing it toward lysosomal degradation (152). Additionally, the renal parenchyma is rich in proteolytic enzymes such as neprilysin (NEP), which degrade circulating Aβ and help lower both plasma levels and brain deposition risk. Emerging studies also indicate that Tau protein may undergo peripheral clearance via renal pathways (148). Although the precise mechanisms remain to be fully elucidated, patients with chronic kidney disease (CKD) frequently present with elevated plasma Tau levels, greater cerebral Aβ and Tau burden, and impaired cognitive function, suggesting a strong link between renal function and Tau clearance capacity (153). In pathological states such as CKD, the kidney’s ability to clear Aβ and Tau is markedly reduced (154). Clinical studies have reported significantly elevated levels of Aβ_40_;, Aβ_42_, and Tau in the plasma of CKD patients, with the degree of brain Aβ deposition inversely associated with renal function (155, 156). These patients are at increased risk of cognitive impairment and dementia. Supporting this, experimental models have demonstrated that surgically induced renal impairment in mice accelerates the accumulation of cerebral Aβ and Tau (157), exacerbating AD-like pathology. Conversely, improvements in renal function—such as through kidney transplantation—can alleviate cognitive deficits, reinforcing the protective role of the kidney–brain axis in AD pathology.

As a non-pharmacological intervention, regular physical exercise has been shown to significantly enhance renal function, thereby facilitating the peripheral clearance of circulating Aβ and Tau proteins, lowering AD risk, and improving cognitive performance. Exercise improves renal hemodynamics by increasing tissue perfusion and oxygen delivery (158), which enhances glomerular filtration and allows for more efficient elimination of Aβ and Tau via urinary excretion. In parallel, it mitigates chronic inflammation and oxidative stress (159), both locally in the kidney and systemically. These pathological states disrupt nephron integrity and upregulate renal expression of the receptor for advanced glycation end-products (RAGE) (160), promoting Aβ retention within renal tissues. Exercise counteracts these effects by reducing levels of pro-inflammatory cytokines such as TNF-α and IL-6, enhancing antioxidant capacity, and thereby limiting RAGE-mediated Aβ accumulation (161). Additionally, long-term exercise suppresses renal fibrosis by downregulating the TGF-β/Smad signaling pathway (162), reducing interstitial collagen deposition and preserving parenchymal integrity. Improvements in systemic metabolic parameters—such as blood pressure, glycemia, and lipid profiles—further support optimal kidney function. Overall, healthy renal function is essential for maintaining peripheral Aβ and Tau homeostasis, while renal impairment may exacerbate toxic protein accumulation and accelerate AD pathology. By enhancing renal filtration, transport, and degradation mechanisms, exercise serves as a comprehensive strategy to promote peripheral clearance of Aβ and Tau and mitigate their neurotoxic impact on the brain.

### 4.4 Exercise enhances glymphatic clearance of Aβ and Tau

**Figure 4:**
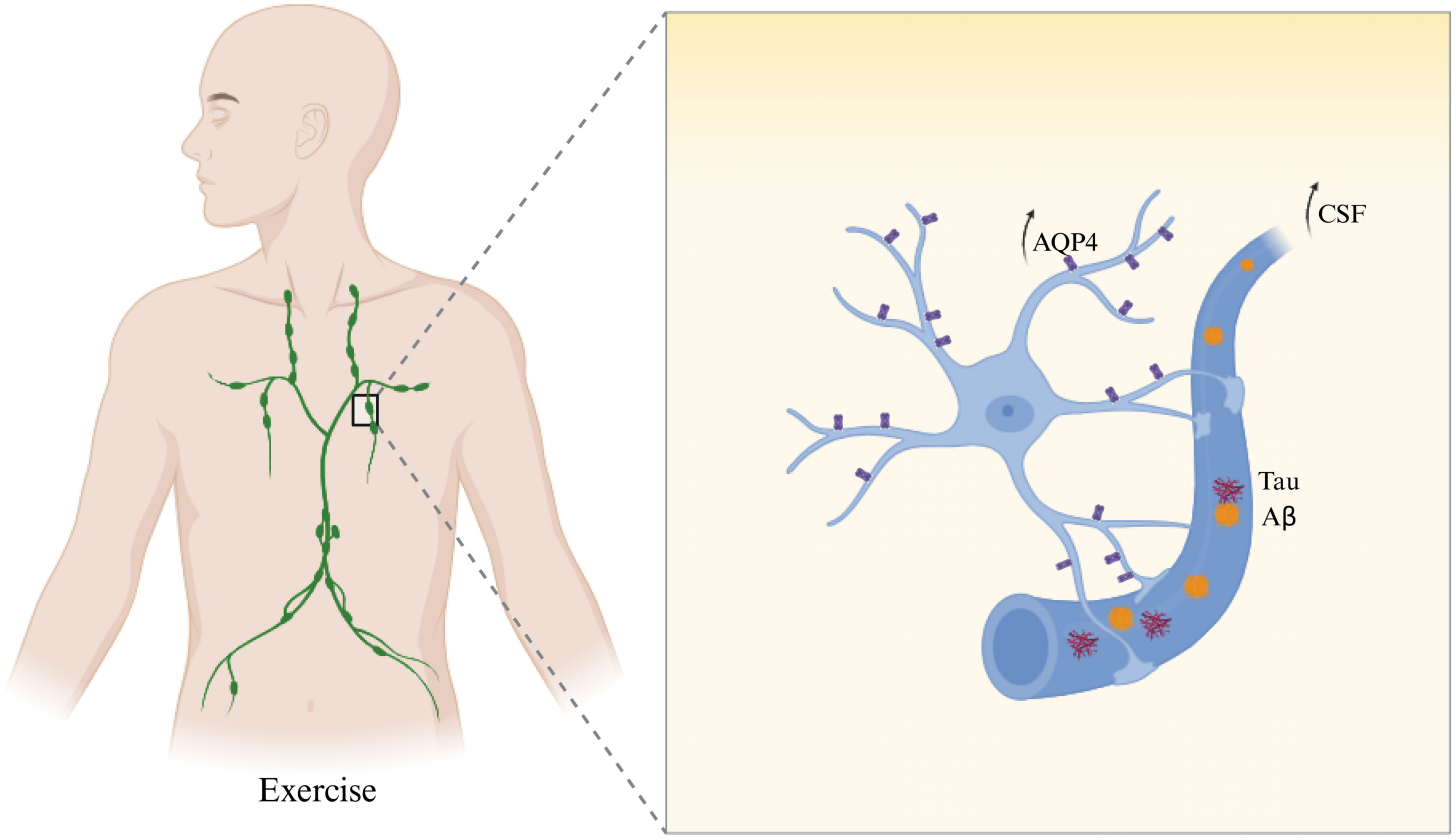
Exercise promotes glymphatic Aβ and Tau clearance via AQP4-mediated CSF–ISF exchange.

The glymphatic system is a key CSF–mediated pathway responsible for clearing metabolic waste from the brain, including pathogenic proteins such as Aβ and Tau (163). Disruption of this system—such as loss of polarized aquaporin-4 (AQP4) expression in astrocytic endfeet or reduced CSF circulation—has been closely linked to the pathogenesis of AD (154). Enhancing glymphatic clearance has thus emerged as a promising therapeutic strategy to mitigate AD progression. Exercise has been shown to activate the glymphatic system and improve brain-to-peripheral waste clearance. One key mechanism is the upregulation and repolarization of AQP4 at astrocytic endfeet, which facilitates CSF–interstitial fluid (ISF) exchange (164). In AD mouse models, aerobic exercise restored AQP4 polarity, enhanced glymphatic flow (165), reduced Aβ deposition and Tau phosphorylation, and improved cognitive performance. These benefits were abolished in AQP4 knockout mice, confirming the essential role of AQP4-mediated glymphatic activity in the neuroprotective effects of exercise. Additionally, exercise promotes fluid exchange between the brain and peripheral lymphatic vessels. Clinical imaging studies indicate that long-term aerobic activity increases CSF flow in the striatum and enhances meningeal lymphatic vessel diameter and velocity (166). These effects reflect enhanced glymphatic–lymphatic connectivity, supporting more efficient clearance of brain-derived toxins. The observed reduction in systemic inflammatory markers post-exercise may further indicate enhanced waste removal via this pathway (16). Furthermore, exercise-induced improvements in sleep architecture also augment glymphatic function. Glymphatic clearance peaks during deep slow-wave sleep, and regular physical activity increases both the duration and quality of this phase (167). As a result, CSF circulation is boosted and nocturnal clearance of Aβ and Tau is enhanced. When combined with healthy sleep hygiene, exercise may synergistically strengthen glymphatic performance and metabolic homeostasis (168). In summary, exercise strengthens glymphatic clearance of Aβ and Tau by restoring AQP4 polarity, enhancing cerebrospinal and lymphatic circulation, and improving sleep-driven clearance rhythms. These combined mechanisms promote efficient brain-to-periphery protein transport, offering a practical and multifaceted intervention to delay or mitigate AD pathology (**Figure 4**).

## 5 Exercise-induced peripheral factors in the regulation of Aβ and Tau homeostasis

As a systemic and multi-target intervention, physical exercise has attracted increasing interest in the context of AD prevention and treatment. Beyond localized effects within the brain, growing evidence suggests that exercise exerts neuroprotective benefits through peripheral factors induced by skeletal muscle contraction (169). These circulating molecules can cross the BBB or signal through systemic pathways to influence Aβ homeostasis within the CNS. The primary mediators of this peripheral regulatory network include myokines, non-coding RNAs (ncRNAs), exosomes, and the metabolic byproduct lactate (170, 171). Together, these components regulate Aβ and Tau clearance, metabolism, and toxicity modulation. Skeletal muscle, as the body’s largest endocrine organ, releases a range of bioactive signaling molecules during physical activity (172). These myokines—secreted via endocrine, paracrine, or exosome-based mechanisms—enter the bloodstream and act on peripheral targets such as the liver, adipose tissue, and the brain. Prominent examples include irisin (173), IL-6 (174), CTSB (175), and glycosylphosphatidylinositol-specific phospholipase D1 (GPLD1) (176), which have been shown to enhance BDNF expression, promote synaptic plasticity, and facilitate Aβ clearance, thereby attenuating AD pathology. Exercise also modulates gene expression through various ncRNAs—including miR-132, miR-124, and miR-146a—that circulate in exosomes and serve as molecular messengers along the muscle–liver–brain axis (170, 177). These ncRNAs regulate inflammation, macrophage polarization, and synaptic stability (178). Furthermore, lactate, a rapidly accumulating byproduct of muscular activity, has emerged as a neuroactive metabolite capable of supporting neuronal energy metabolism, enhancing neurogenesis, and improving synaptic function (80). In summary, elucidating the molecular mechanisms of exercise-induced peripheral signaling may inform the development of exercise mimetics and novel AD therapies aimed at restoring Aβ and Tau homeostasis.

### 5.1 Myokines

Myokines are biologically active peptides synthesized and secreted by skeletal muscle during contraction (179). They have recently been redefined as endocrine messengers that mediate communication between skeletal muscle and distant organs, particularly the brain (180, 181). Beyond its role as a motor effector, skeletal muscle is the body’s largest endocrine organ. During physical activity, myokines are released into the circulation and act through endocrine, paracrine, or autocrine mechanisms to modulate peripheral immune responses, metabolic homeostasis, and inflammatory states—thereby indirectly influencing CNS function (182).

Accumulating evidence indicates that exercise-induced myokines contribute to neuroprotection in AD by regulating systemic inflammation, lipid metabolism, neurotrophic factor expression, and peripheral clearance of Aβ (123, 180). Interleukin-6 (IL-6) was the first identified myokine, robustly secreted by contracting skeletal muscle during exercise (183). IL-6 activates its receptor pathway to induce the expression of anti-inflammatory cytokines (e.g., IL-10, IL-1ra) while inhibiting pro-inflammatory mediators such as TNF-α (184), thereby establishing a systemic anti-inflammatory environment (185). Reduced peripheral IL-6 expression is commonly observed in AD patients, suggesting early impairment in this myokine-mediated regulatory axis. However, this reduction is likely multifactorial and may also reflect the effects of sarcopenia, immune dysfunction, and comorbid conditions (186–188). Regular exercise restores IL-6 levels (189), improves immune balance, and indirectly attenuates neuroinflammation and Aβ accumulation (190). IL-6 also influences lipid metabolism and oxidative stress, further supporting peripheral Aβ clearance.

Irisin, another well-studied myokine, is generated through cleavage of the FNDC5 precursor protein under the control of PGC-1α (191). Circulating irisin binds to integrin αVβ5 receptors (192), activating the AMPK–BDNF axis to enhance neurotrophic support (193), modulate immune cell activity, and reduce peripheral inflammation. Blocking systemic irisin expression significantly attenuates the cognitive benefits of exercise (103, 194), underscoring its essential role in mediating exercise-induced neuroprotection.

CTSB, a lysosomal cysteine protease, is markedly elevated in the bloodstream following exercise (195). CTSB enhances peripheral BDNF expression and contributes to anti-inflammatory responses and Aβ clearance (187). It is hypothesized to function as a key mediator in the muscle–liver–brain axis. Notably, CTSB-deficient mice fail to exhibit exercise-induced cognitive improvements, highlighting its necessity for trans-systemic neuroprotection.

GPLD1, primarily synthesized in the liver, is also upregulated following exercise (176). It regulates signaling pathways via cleavage of GPI-anchored proteins and promotes peripheral neurotrophic factor production (196). Circulating GPLD1 has been shown to mediate exercise-induced cognitive benefits even in sedentary conditions, reinforcing its role in peripheral-to-central signaling.

Clusterin (Clu, also known as ApoJ) is another exercise-responsive factor (15). Peripheral Clu expression increases after physical activity and facilitates Aβ binding and efflux from the brain (15). Clu also modulates the complement system and suppresses peripheral inflammation, indirectly supporting neuronal function (197). Moreover, Clu upregulation correlates with peripheral neural progenitor cell proliferation and may promote overall neural plasticity. However, Clu’s role appears context-dependent; under certain inflammatory conditions, it may promote Aβ aggregation, suggesting a dualistic or conditional regulatory role (198). In summary, myokines serve as crucial messengers linking physical activity to peripheral and central signaling. Through modulation of immune, metabolic, inflammatory, and Aβ clearance pathways, they contribute to maintaining Aβ and Tau homeostasis in the brain. Understanding the peripheral mechanisms and interactive networks of myokines offers novel insights into the molecular basis of exercise therapy and may inform the development of myokine-based therapeutic strategies for AD.

### 5.2 Non-coding RNAs and exosomal factors

Non-coding RNAs, particularly microRNAs (miRNAs), are increasingly recognized as peripheral signaling mediators with relevance to AD (199). These approximately 22-nucleotide-long RNAs regulate gene expression at the post-transcriptional level, primarily by binding to complementary sequences in target mRNAs to promote their degradation or translational repression, and circulate freely or within exosomes to mediate peripheral–central communication (200). Exercise profoundly alters miRNA expression profiles and promotes the expression of neuroprotective miRNAs that modulate inflammation, Aβ metabolism, neurotrophic signaling, and BBB integrity (201). Exercise-induced upregulation of miR-126 and miR-146a improves cerebrovascular health and limits Aβ accumulation by enhancing endothelial function and reducing inflammation (202, 203). Meanwhile, downregulation of pro-inflammatory miR-155 and upregulation of neuron-specific miR-124 help reprogram peripheral and central immune responses (178, 204). In parallel, liver-expressed miR-29 family members downregulate BACE1 expression, thereby reducing peripheral Aβ generation (79). Importantly, exosomes derived from exercised animals, enriched in specific miRNAs, have been shown to reduce brain Aβ levels and improve cognition in AD models, suggesting a coordinated miRNA–exosome–periphery–CNS axis (205). These findings position miRNAs as both biomarkers and therapeutic targets for AD. Continued research into their dynamic expression, molecular targets, and delivery mechanisms will be essential for translating exercise-based interventions into clinical strategies.

### 5.3 Lactate

Lactate has long been viewed as a metabolic waste product of anaerobic glycolysis during intense physical activity, often associated with muscle soreness and fatigue (206). However, this traditional perspective has been fundamentally challenged in recent years. Lactate is now recognized as a hormonally active metabolic signal—termed a “lactormone”—that mediates inter-organ communication and plays vital roles in metabolic regulation and signal transduction across multiple systems (207, 208). Beyond its role in energy redistribution, lactate also influences gene expression, protein synthesis, and intercellular signaling, with broad physiological implications. In the CNS, lactate can cross the BBB and is taken up by neurons and astrocytes (209), where it serves as both an energy substrate and signaling molecule. It enhances synaptic plasticity, increases cerebral blood flow, induces the expression of BDNF, and participates in epigenetic regulation such as histone acetylation (210). In neurodegenerative diseases such as AD, lactate has demonstrated neuroprotective properties. Exogenous lactate administration has been shown to elevate hippocampal BDNF expression (211), improve cognitive performance in AD models, suppress neuroinflammation, reduce Aβ accumulation (212), and attenuate Tau hyperphosphorylation (213). These effects are mediated, at least in part, via activation of the GPR81 lactate receptor and the NAD^+^/SIRT1 signaling axis within the hippocampus and other CNS tissues (80, 214). Importantly, exercise is the principal physiological source of lactate. During moderate to intense aerobic activity, skeletal muscle produces and releases large quantities of lactate into circulation, establishing a “muscle–lactate–brain” signaling axis. Circulating lactate can directly reach the brain or exert effects on peripheral organs that secondarily influence CNS homeostasis. For example, lactate upregulates metabolic regulators such as PPARγ and SIRT1 in the liver and adipose tissue (215), thereby improving lipid metabolism and insulin sensitivity—both of which are linked to AD risk reduction. Concurrently, lactate promotes the polarization of peripheral macrophages toward an anti-inflammatory M2 phenotype and inhibits the release of pro-inflammatory cytokines (216, 217), thus alleviating chronic systemic inflammation relevant to AD pathology. Moreover, lactate facilitates metabolic reprogramming between muscle and liver, inducing the expression of hepatokines such as GPLD1 and FGF21 (218), which have been shown to cross the BBB and exert neuroprotective effects. Exercise not only enhances lactate production but also improves its systemic clearance and buffering capacity, sustaining its function as a long-range signaling molecule. In AD mouse models, treadmill training elevates plasma lactate levels, which act as a mechanistic mediator to enhance hippocampal BDNF expression, reduce Aβ deposition, and improve cognitive function (219). These benefits are significantly diminished when monocarboxylate transporters (MCTs) (220), responsible for lactate transport, are pharmacologically inhibited—highlighting lactate’s indispensable and independent role in mediating exercise-induced neuroprotection. In summary, lactate acts as a central metabolic mediator linking exercise with brain health. Through its capacity to modulate systemic metabolism, immune function, neurotrophic support, and energy redistribution, lactate orchestrates a multi-tiered “exercise–periphery–brain” regulatory pathway that holds therapeutic potential for delaying or mitigating AD pathology.

## 6 Conclusion

The central pathological hallmark of AD is the disruption of cerebral metabolic homeostasis of Aβ and Tau proteins (181, 200). This imbalance is governed not only by intrinsic mechanisms within then CNS, but also by dynamic and coordinated regulatory processes in peripheral systems. In this review, we have summarized emerging evidence demonstrating how physical exercise maintains brain Aβ and Tau homeostasis through peripheral modulation of inflammation, immunometabolic networks, clearance mechanisms, and systemic signaling. These findings underscore the pivotal role of the “exercise–periphery–brain” axis as a systemic regulatory framework. Exercise acts as a key integrator linking peripheral and central compartments, and the exercise-induced peripheral mediators—such as myokines, miRNAs, exosomes, and lactate—exert hormone-like, multitarget, and long-range effects critical for modulating AD pathology.

Despite these advances, important scientific questions remain. Do different peripheral factors act synergistically or independently? How is their inter-organ transport and spatial selectivity orchestrated? How do these factors selectively affect neurons, glial cells, or the BBB? Addressing these questions is essential for deciphering the mechanistic logic of peripheral–central communication in neurodegeneration. Future research should incorporate integrative multi-omics platforms—including single-cell and spatial transcriptomics, proteomics, and metabolomics—combined with functional validation via gene editing, transgenic models, and translational models such as humanized AD mice or 3D brain organoids. These tools will enable fine-resolution mapping of the dynamic regulation of the exercise–periphery–brain axis across disease stages, and identify organ- or time-specific therapeutic targets.

Given their pharmacological potential, exercise-induced factors are also fueling the development of “exercise mimetic interventions.” Translating agents such as exosomes, miRNAs, and irisin into clinically usable, dose-controllable, and CNS-targeted therapeutics may provide alternatives for individuals unable to participate in regular physical activity. In this context, advances in molecule purification, delivery vector design, and brain-targeted transport strategies will be critical for moving toward next-generation neurotherapeutics.

Lastly, we highlight the importance of accounting for individual variability in exercise responsiveness. Biological factors such as sex, age, genetic background, and metabolic status significantly influence the expression profiles and CNS impact of peripheral mediators (201, 202). Personalized intervention programs—anchored in physiological diversity—may facilitate the development of precision exercise-based therapies. Moving forward, interdisciplinary collaboration among basic science, clinical neurology, and biomedical engineering will be pivotal in driving the translation of systemic exercise-based strategies into effective early interventions for AD.
